# Association between tooth agenesis and cancer: a systematic review

**DOI:** 10.1590/1678-7757-2020-0955

**Published:** 2021-08-09

**Authors:** Melany Clarissa Gámez Medina, Renata Travassos da Rosa Moreira Bastos, Paulo Mecenas, João de Jesus Viana Pinheiro, David Normando

**Affiliations:** 1 Universidade Federal do Pará Programa de Pós-Graduação em Odontologia BelémPará Brasil Universidade Federal do Pará, Programa de Pós-Graduação em Odontologia, Belém, Pará, Brasil.; 2 Universidade Federal do Pará Faculdade de Odontologia BelémPará Brasil Universidade Federal do Pará, Faculdade de Odontologia, Belém, Pará, Brasil.

**Keywords:** Tooth agenesis, Neoplasm, Cancer, Anodontia

## Abstract

The congenital absence of multiple teeth may share the same genetic background of the development of some types of cancer. Objective: This systematic review aimed to investigate the possible association between dental agenesis and cancer, and the perspective of agenesis as an early predictor for cancer risk. Methodology: The electronic databases PubMed, Scopus, Web of Science, Cochrane Library, LILACS, and OpenGrey were searched and the risk of bias was evaluated using the Newcastle-Ottawa tool. The GRADE tool was used to evaluate the certainty of the evidence. Results: Six studies met the eligibility criteria. A positive co-occurrence between ovarian cancer and hypodontia was found in two articles. Three studies evaluated the association between dental agenesis and colorectal cancer and only one showed common genes for these conditions. One paper found individuals with hypodontia had a higher risk of family history of cancer. Five studies had a fair quality and one a good quality. The certainty of evidence was classified as very low. Conclusion: Notwithstanding the limited scientific evidence, there may be a possible association between dental agenesis and cancer due to genes involved in both conditions. Agenesis of multiple teeth could be an early indicator of cancer risk. Nevertheless, studies with a better level of evidence are needed to confirm this possible association.

## Introduction

Tooth agenesis is a common dental anomaly in humans, with prevalence around 6.4% and variation according to sex, race, and ethnicity.^1^ Hypodontia is the condition characterized by the absence of less than six permanent teeth, oligodontia more than six missing teeth, and anodontia in case of missing all permanent teeth.^2^^,^^3^ Except for the third molars, lower second premolars and upper lateral incisors are the permanent teeth most affected.^1^^,^^4^

Some environmental factors could interfere with odontogenesis, which includes trauma, infection, smoking, surgery, and others.^5^^-^^8^ Tooth agenesis is also related with genetic syndromes including ectodermal dysplasia and Klinefelter syndrome.^9^ Therefore, this condition may also be classified as syndromic or non-syndromic.^4^^,^^10^ In both situations, the genetic seems to be the main etiological component.^11^

Mutations and single nucleotide polymorphism (SNP) in some genes, such as axis inhibition protein 2 (AXIN2),^12^ muscle segment homeobox 1 (MSX1),^13^ paired box gene 9 (PAX9),^14^ and wingless type MMTV integration site family, member 10A (WNT10A)^15^ have been related with dental agenesis and interestingly, mutations in these genes may be connected with many types of cancers.^16^

The link between dental agenesis and cancer may be elucidated by three factors: (1) there are genes involved in odontogenesis that are present in tumor tissues or cells;^17^^,^^18^ (2) nucleotide changes on some genes are related with both odontogenesis and cancer^19^ and the mutations appear to disturb odontogenesis early in life and later contribute to the carcinogenesis; (3) according to epigenetics, the aberrant methylation of these genes was observed in neoplasm samples.^20^

There is still divergence in the literature about this relationship. Some studies showed an association between dental agenesis and cancer,^21^^,^^22^ while others do not.^23^^,^^24^ There is a great clinical relevance in this issue since the absence of multiple teeth may be an indicator of cancer. Therefore, this systematic review aims to verify the connection between dental agenesis and cancer, considering a single tooth agenesis or even oligodontia, and the possibility that agenesis is an early indicator for cancer risk.

## Methodology

This review was registered at PROSPERO database (CRD42019129901) and performed according to PRISMA guidelines.^25^ The process was performed separately by two reviewers. A third reviewer was consulted when there was no agreement between the two reviewers.

### Eligibility criteria

The following eligibility criteria were adopted in this systematic review in accordance to the PECOS format: Population (P): humans; Exposure (E): any type of tooth agenesis; Comparison (C): absence of tooth agenesis; Outcome (O): any type of cancer or family history of cancer; Study design (S): case-control, cross-sectional, or cohort. Studies which evaluated syndromic patients, cases of tooth extraction, patients with cleft lip and palate and third molar agenesis were excluded, as well as opinion articles, animal studies, laboratory studies, case reports, case-series, and literature reviews.

### Information sources

The databases PubMed, Scopus, Web of Science, Cochrane Library, OpenGrey, and LILACS were searched between the 15^th^ and 21^st^ of January and the alerts were followed up until the 5^th^ of September. A manual search was carried out in the reference list of the included studies for eventual relevant article missed during the searches. No restriction on language or publication date was used.

### Search strategy and study selection

**Figure 1:**
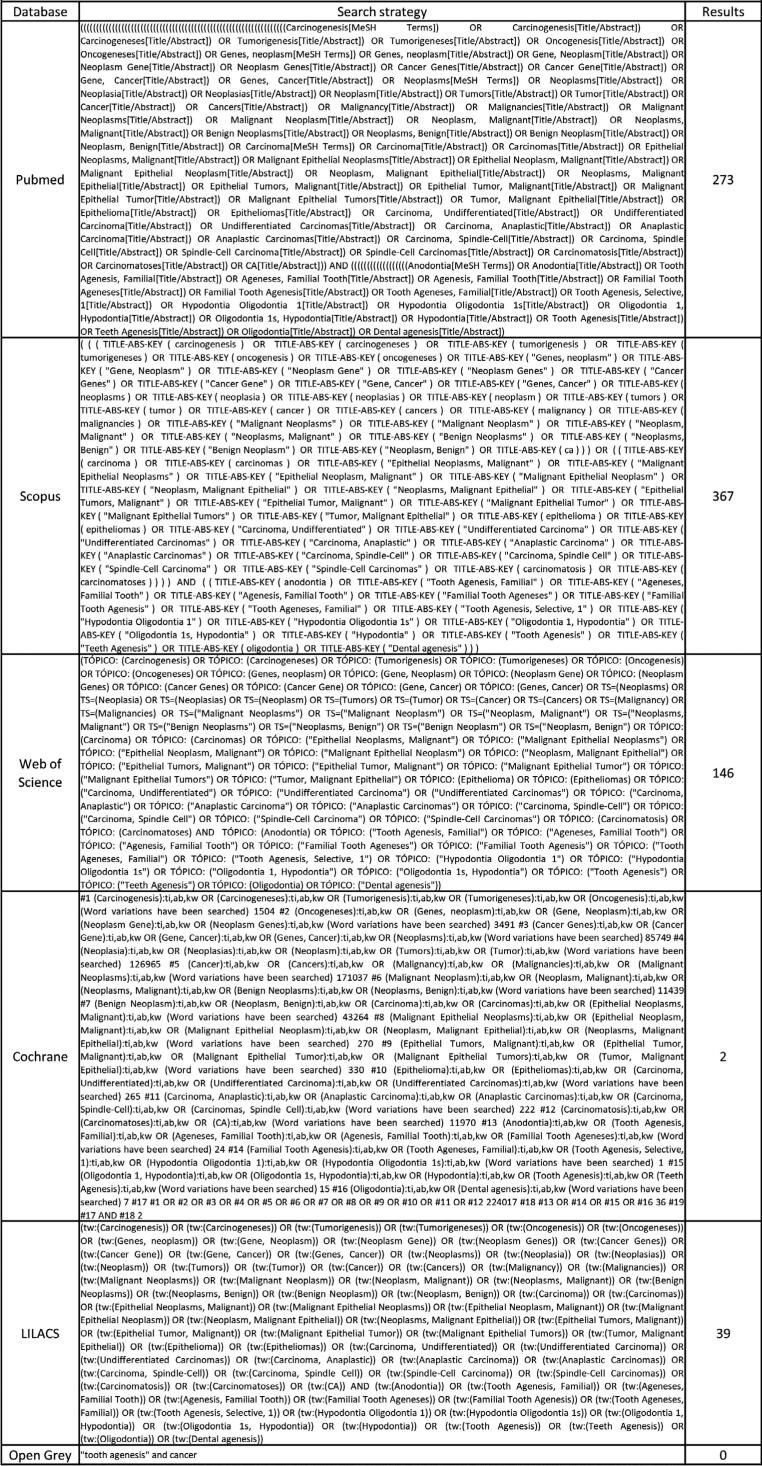
Search strategy

The search strategy was created using words associated with the PECOS strategy and these words were combined using Boolean operators. The search strategy for each database is presented in Figure 1. All relevant references have been imported into the software Endnote (x9 version, Clarivate Analytics, Philadelphia, PA, USA). After duplicate removal, titles, and abstracts were evaluated considering the selection criteria. The included studies were accessed by full text read for further assessment and data extraction.

### Data extraction

The following information was extracted from the selected articles: author, year, country, study design, type of cancer, total cases/controls, incidence of tooth agenesis, mean age, most frequent missing teeth, family history of cancer, genetic analysis, evaluation method, statistical analysis, and main results.

### Risk of bias in individual studies

The Newcastle-Ottawa toll was used to assess the risk of bias.^26^ All studies were evaluated by eight items, grouped into three domains: selection of the study groups, comparability of groups and exposure or outcome assessment for case-control, and cohort studies, respectively. One star was awarded for each quality item, with a maximum of nine stars for the highest quality studies. If the score was eight or more stars the study was classified as “good,” between five and seven as “fair,” equal or less than four as “poor.”

### Summary measurements

The difference in prevalence rates of tooth agenesis between control and case groups was determined by using the p-value <0.05. The association was calculated through the Odds Ratio with a 95 percent confidence interval.

### Certainty of evidence

The certainty of evidence was assessed using the Grading of Recommendations, Assessment, Development, and Evaluation Pro software (GRADE) (GRADEpro, gradepro.org.).^27^ The GRADE analyzes five domains to classify the certainty of evidence: type of study, risk of bias, consistency, directness, and precision of the articles. The certainty of evidence was rated as high, moderate, low, or very low. The outcomes assessed were: “association between tooth agenesis and ovarian cancer,” “association between tooth agenesis and colorectal cancer” and “association between tooth agenesis and family history of cancer.”

## Results

### Study selection

**Figure 2:**
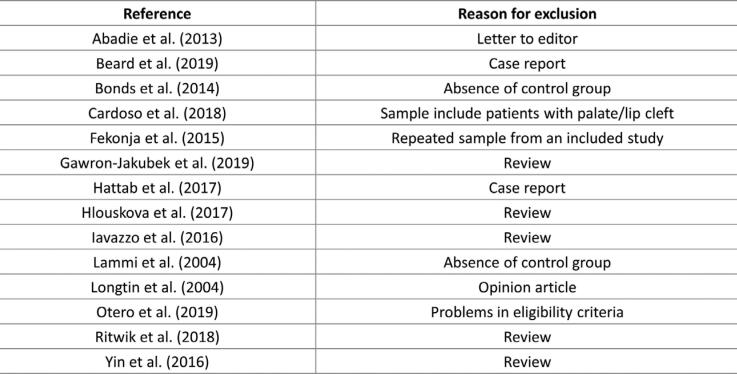
List of excluded studies with reasons for exclusion

**Figure 3:**
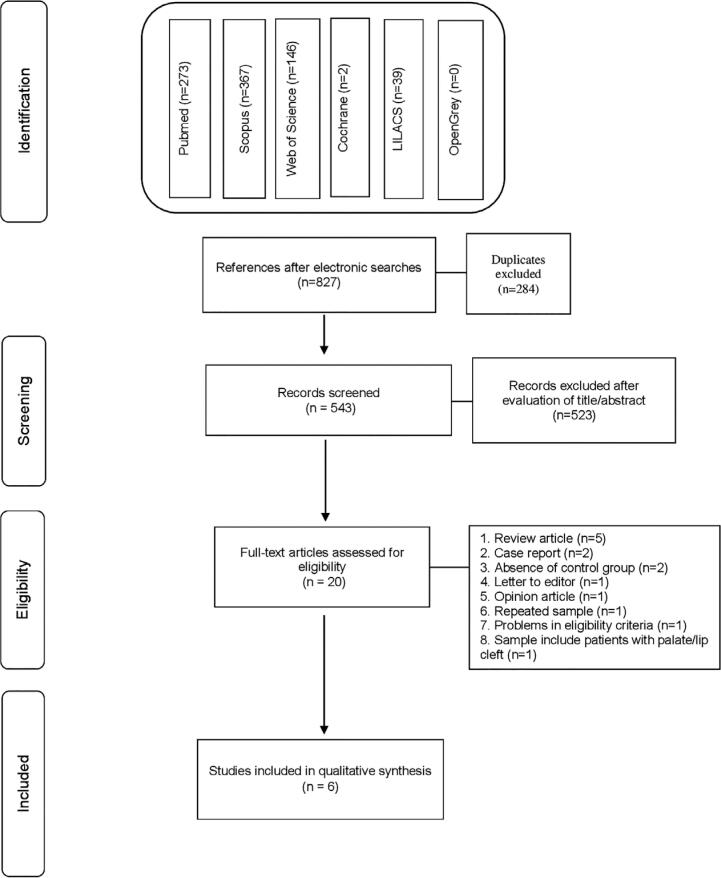
Flowchart with number of records at each stage of the review according to PRISMA statement

The electronic screening found 827 articles: 273 from PubMed, 367 from Scopus, 146 from Web of Science, two from Cochrane Library, 39 from LILACS, and zero from OpenGrey. After removing duplicates studies, 543 articles were identified. After the authors performed title and abstract screening, 20 articles were assessed by full text. Among them, 14 were excluded for the reasons shown in Figure 2. Finally, six studies were selected for qualitative analysis of risk of bias (Figure 3).

### Characteristics of included articles

The characteristics of the six included articles are presented in Table 1. They were observational and case-control studies.^21^^,^^22^^,^^24^^,^^28^^-^^30^ Two articles investigated the association between tooth agenesis and ovarian cancer,^21^^,^^22^ whereas three assessed the interrelation of colorectal cancer with tooth agenesis.^24^^,^^29^^,^^30^ One article investigated the co-occurrence of dental agenesis and family history of cancer.^28^ One of the studies that investigated the relationship between agenesis and colorectal cancer also identified family history of cancer; nonetheless, it was not statistically tested or discussed in the manuscript.^30^ Therefore, we decided to perform an Odds Ratio to assess this association.

**Table 1:** Summary of the data from the studies included in this review

Authors, year, location and type of study	Type of cancer	Sample		Mean Age (years)	Most frequent missing tooth	Family history of cancer control/ case (%)	Genes	Evaluation method	Statistical analysis	Main results
		Without tooth agenesis (with cancer or family history)	With tooth agenesis (with cancer or family history)							
Chalothorn et al.,^21^ (2008), USA, Case-control	Epithelial Ovarian cancer (EOC)	127 (30)	23 (20)	–	U2, U5	–	–	–	Fisher exact, OR	Possible association between EOC and agenesis. The data also showed that the crude OR was 8.1 (95 percent CI, 2.1-30.9), which implied that women with EOC are 8.1 times more likely to have hypodontia than are women without EOC.
Fekonja et al.,^22^ (2014), Slovenia, Case-Control	Epithelial Ovarian cancer (EOC)	209 (97)	31 (23)	–	U5, U2, L5, L1	–	–	Clinical and X-rays evaluations	t-test, X^2^, Fisher exact, OR	The difference between the two groups was statistically significant (p=0.004); the crude OR was 3.30 (95% CI, 0.12–7.01). Women with ovarian cancer were 2.87 times (19.2%–6.7%) more likely to have hypodontia than healthy women.
Paranjyothi et al.,^24^ (2018), India, Case-control	Colorectal cancer (CRC)	44 (21)	6 (4)	–	L5, U2, U5	–	–	Self-reported questionnaire,clinical and X-rays evaluations	X^2^	Sixteen percent of cancer patients and 8% of individuals without cancer reported having tooth agenesis, no statistical difference (p= 0.384).
Lindor et al.,^29^ (2014), Canada, Case-control	Colorectal cancer (CRC)	4188 (1558)	236 (78)	–	–	–	–	Self-reported questionnaire	X^2^, Fisher exact test	4.8% of cases and 5.7% of controls reported having at least one missing tooth, no statistical evidence of difference (p= 0.20).
Williams et al.,^30^ (2018), USA, Case-control	Colorectal cancer (CRC) and family history of any type of cancer	347 (39)	93 (28)	–	–	11,2%/30,1%	ATF1, DUSP10, CASC8	Clinical and radiographic examinations and associated gene variants	–	Genome-wide significant associations were found between TA and ATF1 (P = 4.36 × 10−10) and DUSP10 (P = 1.25 × 10−9), and positive association found with CASC8 (P = 8.2 × 10−5).
Küchler et al.,^28^ (2013), USA/Brazil, Case-control	Family history of any type of cancer	328 (102)	82 (45)	Case:18.15 ± 10.2 Control: 20.33 ±14.9	U4, L4, U5, L5, U2, L1, L2	31,1%/54,90%	AXIN2, FGF3, FGF10, FGFR2	Structured questionnaire and clinical and X-rays evaluations	Student's t test, OR, X^2^, Fisher's exact tests	Individuals with tooth agenesis had an increased prevalence of having a family history of cancer OR = 2.7; 95% C.I., 1.6-4.4). A significant association between AXIN2, FGF3, FGF10, and FGFR2 and tooth agenesis was found.

U2 - Upper lateral incisor; L1 - Lower central incisor; U4/5 - Upper premolars; L4/5 - Lower premolar, CRC: colorectal cancer; AXIN2: Axis inhibition protein 2.

A considerable difference was found in relation to the sample sizes. The sample sizes of the control groups ranged from 44^24^ to 4188,^29^ while the sample sizes for the case groups ranged from 6^24^ to 236.^29^ The mean age was only reported by one article.^30^ The diagnosis of dental agenesis was made through clinical,^21^^,^^22^^,^^24^^,^^28^^,^^30^ radiographic examination,^21^^,^^22^^,^^24^^,^^28^^,^^30^ and a self-report questionnaire.^24^^,^^29^ The tooth with the highest percentage of congenital absence were upper lateral incisor,^21^^,^^22^^,^^24^^,^^28^ second upper premolars,^21^^,^^22^^,^^24^ second lower premolars,^22^^,^^24^ and lower central incisors.^22^ The diagnosis of cancer was not detailed in the studies, although they report patients were diagnosed and recruited from cancer treatment centers.^21^^,^^22^^,^^24^^,^^29^ The family history of cancer was evaluated in the included studies through questionnaires^28^ or self-reports.^30^ Two studies^28^^,^^30^ evaluated the relation through genes analysis, which were: AXIN2, FGF3, FGF10, FGFR2,^28^ ATF1, DUSP10, CASC8.^30^

### Results of individual studies

Two studies detected an association between the congenital absence of tooth and ovarian cancer.^21^^,^^22^ Other two articles did not report an association between colorectal cancer and dental agenesis,^24^^,^^29^ whereas one showed common genes for both conditions: ATF1, DUSP10, and CASC8.^30^ One study found subjects with dental agenesis had a major chance of family history of cancer and associations with AXIN2, FGF3, FGF10, and FGFR2 genes.^28^

### Synthesis of results

It was not possible to perform a meta-analysis because of the low number of articles investigating the analyzed outcomes. However, an odds ratio was performed for each study individually and for each type of cancer or family history of cancer. It was revealed a statistically significant association between dental agenesis and ovarian cancer, with a chance of a patient with ovarian cancer being diagnosed with tooth agenesis 6.43 higher. No statistically significant association was observed between agenesis and colorectal cancer, which is corroborated by the p-value and the 95% confidence interval. Finally, a statistically significant association was also noticed between family history of cancer and dental agenesis and the results shows a chance 2.71 times greater of the co-occurrence of these two conditions (Table 2).

**Table 2:** Odds ratio of the included studies

Cancer type or family history	Study	Group	With cancer or family history	Without cancer or family history	Total	OR/95CI (Study)	p-value (study)	OR/95CI (each cancer type or family history)	p-value (each cancer type or family history)
Ovarian cancer	Chalothorn et al.,^21^ (2008)	With agenesis	20	3	23	OR: 21.56/5.99-77.58	<0.0001	OR: 6.43/ 3.20- 12.93	<0.0001
		Without agenesis	30	97	127				
	Fekonja et al.,^22^ (2014)	With agenesis	23	8	31	OR: 3.32/1.42-7.76	0.0070		
		Without agenesis	97	112	209				
Colorectal cancer	Paranjyothi et al.,^24^ (2018)	With agenesis	4	2	6	OR: 2.19/0.36-13.22	0.6634	OR: 0.86/ 0.66- 1.13	0.3151
		Without agenesis	21	23	44				
	Lindor et al.,^29^ (2014)	With agenesis	78	158	236	OR: 0.83/0.63-1.10	0.2240		
		Without agenesis	1558	2630	4188				
Family history of cancer	Williams et al.,^30^ (2018)	With agenesis	28	65	93	OR: 3.40/1.95-5.92	<0.0001	OR: 2.71/ 1.90- 3.86	<0.0001
		Without agenesis	39	308	347				
	Küchler et al.,^28^ (2013)	With agenesis	45	37	82	OR: 2.69/1.64-4.4155	0.0001		
		Without agenesis	102	226	338				

### Risk of bias assessment

The quality of five studies was classified as fair,^22^^,^^24^^,^^28^^-^^30^ and one study as good (Table 3).^21^ Limitations were found in the main domains evaluated. The domain “selection of study groups” exhibited deficiencies such as inadequate case definition;^29^ poor representativeness of the cases^21^^-^^24.28^^-^^30^, and lack of information on the selection of controls.^22^^-^^24^^,^^29^ The deficiency in the representativeness of the cases was characterized by no description of the recruitment location of control subjects. The domain “comparability of groups” presented limitations in the item “no control of important confounding factors (e.g. gender, age).”^24^ Two articles showed inaccurate outcome assessments due to evaluation by self-reporting.^28^^,^^30^

**Table 3:** Risk of Bias of the studies, according to the Newcastle-Ottawa Scale

Study	Selection (maximum 4 stars)	Comparability (maximum 2 stars)	Outcome or exposure assessment (maximum 3 stars)	Total score (Quality)
Chalothorn et al.,^21^ (2008)	3	2	3	8 (Good)
Fekonja et al.,^22^ (2014)	2	2	3	7 (Fair)
Paranjyothi et al.,^24^ (2018)	2	1	3	6 (Fair)
Lindor et al.,^29^ (2014)	1	2	3	6 (Fair)
Küchler et al.,^28^ (2013)	3	2	2	7 (Fair)
Williams et al.,^30^ (2018)	3	2	2	7 (Fair)

### Level of evidence

**Figure 4:**
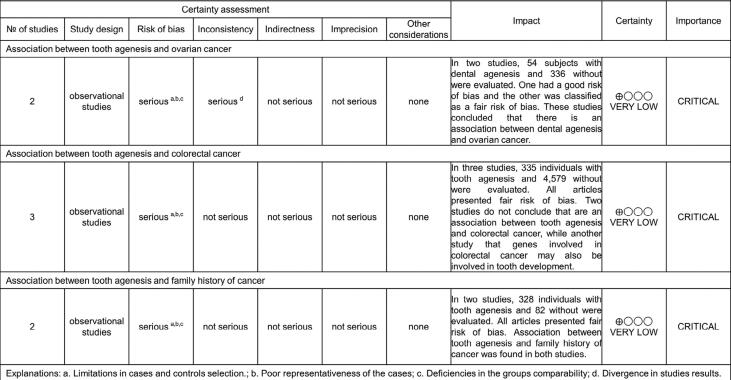
Grade evidence profile table

The GRADE evaluation found a very low certainty of evidence for the three outcomes assessed (Figure 4). This can be associated with the study design and risk of bias of included articles.

## Discussion

Odontogenesis is an intricate process of reciprocal interaction with the involvement of a larger number of genes and the opportunity of mutations in many of these genes can disrupt this process and be associated with hypodontia.^22^^,^^28^ The genes that command teeth development also have important functions and molecular association with other organs and body systems. Therefore, a genetic alteration culminating in hypodontia can lead to abnormalities in other parts of the human body.^21^ Some selected articles in this systematic review point to a potential association between dental agenesis and neoplasm.^19^^,^^21^^,^^28^^,^^31^^-^^33^

One of the most related genes to dental agenesis is AXIN2. The protein expressed by this gene has an important function in craniofacial morphogenesis.^16^ Patients with SNP of the AXIN2 gene do not have permanent molars, premolars, lower incisors, and upper incisors.^18^ Interestingly, mutations in the AXIN2, MSX1, PAX9, and WNT10A genes may be associated with cancers.^16^ This condition refers to a phenomenon called pleiotropy, characterized by a single genetic locus that truly affects multiple apparently unrelated phenotypic traits. It is often identified as a single mutation that affects two or more wild-type traits in human complex diseases that share the same genetic pathways.^34^^,^^35^

### Summary of evidence

Six final articles were screened in this systematic review, and methodological characteristics were analyzed. In relation to the classification of the articles using the Newcastle-Ottawa tool, five studies were classified as fair quality,^22^^,^^24^^,^^28^^-^^30^ and one as good^21^ due, among other factors, to poor representativeness of the cases. The GRADE tool was used for the assessment of the certainty of evidence. A very low certainty of evidence was scored because of the study designs, and the result obtained in the assessment of risk was biased.

In two included studies, the authors verified the association between dental agenesis and ovarian cancer.^21^^,^^22^ Chalothorn, et al.^21^ (2008) used dental and medical records to assess family history of cancer and tooth agenesis. The dental examination was conducted to detect clinically hypodontia or any phenotype involved with this congenital dysfunction, like microdontia and agenesis. As a result, the authors found an increased prevalence of tooth agenesis in patients with epithelial ovarian cancer. In another study, conducted by Fekonja, Čretnik, and Takač^22^ (2014) women diagnosed with epithelial ovarian cancer were evaluated through clinical examination and panoramic radiography to confirm the diagnosis of hypodontia. The results showed a possible association between the two conditions. The OR confirmed a significant association between ovarian cancer and tooth agenesis. The result indicated the chance of a patient with ovarian cancer be diagnosed with a pattern of dental agenesis is 6.43 times greater (Table 2).

The findings of these two studies^21^^,^^22^ differ from other results in the literature that point to independent causation of these conditions.^23^ The authors analyzed the ovarian cancer sample in a cohort study and do not prove that the two conditions are independent from each other, but a genetic connection between them needs more epidemiological studies and molecular analysis to be confirmed. The absence of an adequate control group definition did not allow its inclusion in this systematic review.^23^

Regarding the association between dental agenesis and colorectal cancer, one^29^ of the three included articles^24^^,^^29^^,^^30^ used a questionnaire to self-report information on congenitally missing teeth. This was a limitation since a dental clinician did not examine the participants, and therefore justified the fair quality rating. The authors concluded the study did not provide scientific evidence strong enough to prove the predisposition of dental agenesis among colorectal cancer patients. The second study^24^ which verified this association agrees with the results obtained by Lindor, et al.^29^ (2014). The patients with colorectal cancer revealed an increased prevalence of dental agenesis when confronted to patients without history of this cancer, but it was not statistically significant. Our OR results, as well, demonstrated no statistically significant association between the two conditions, as demonstrated by the p-value and 95% confidence interval (Table 2).

The major contrast from this study^24^ to the Lindor, et al.'s^29^ (2014) was the clinical and radiographic analysis of tooth agenesis, which was performed by the same dentist to avoid interexaminer bias, in the first,^24^ compared to a self-reported questionnaire of hypodontia in the second.^29^

In the third included article,^30^ the dental diagnosis was made by a dentist through clinical and radiographic exams. This study carried out a genetic analysis for which the authors selected 30 colorectal cancer-predisposing single nucleotide variants with genome-wide significance. The authors concluded the genes related with colorectal cancer may also be involved in odontogenesis, and it provides extra perception into clarifying complex etiology and association between colorectal cancer and hypodontia. Furthermore, they found new genes and gene pathways continue with an unknown role in relation to tooth development.

Studies showed an increased presence, in patients with congenital missing teeth, of cancer in relatives^29^^,^^30^ and a genetic link would be manifested more strongly in first-degree relatives.^36^ To verify this relationship, Küchler, et al.^28^ (2013) studied the family history of cancer and its co-occurrence with tooth agenesis, corroborating the hypothesis that both conditions share a similar genetic background, with an increased overall cancer occurrence between relatives of people with tooth agenesis. Over a decade ago, Lammi, et al.^19^ (2004) first visualized a genetic alteration in the AXIN2 gene that causes both situations in a large multiplex family. The results obtained with OR showed a significant association between tooth agenesis and a family history of cancer, being the chance of a patient with family history of cancer being diagnosed with tooth agenesis 2.71 times higher (Table 2).

Dental agenesis is a failure in the odontogenesis process that occurs at the beginning of tooth morphogenesis.^37^ It is well known the etiology is related with genetic and environmental factors,^38^ and it may be part of a phenotypic expression of a syndrome or occur in isolation.^37^ The genes that are often associated with non-syndromic dental agenesis are AXIN2, MSX1, PAX9, EDA, and WNT10.^39^^,^^40^

It has been reported the association of AXIN2 gene with colorectal cancer.^19^^,^^33^^,^^41^ However, this relationship has not been demonstrated yet,^42^^,^^43^ which corroborates the results of this systematic review. In consequence, the polymorphism in AXIN2 gene may be considered a biological risk marker for predisposition and prognosis of colorectal cancer.^41^ A possible genetic relationship between dental agenesis and colorectal cancer has also been studied by Williams, et al.^30^ (2018) which reported the ATF1, DUSP10 and CASC8 genes may be related to colorectal cancer and to odontogenesis.

The hypothesis of tooth agenesis as a risk factor can be considered when evaluated the association with ovarian cancer, helping in its the early detection. In this case, however, it was not found an inherent gene that might be the causal factor responsible for the connection between the two conditions, as recently reported in the literature.^44^ The genes BRCA1 e BRCA2 are the strongest recognized genetic risk factors for epithelial ovarian cancer,^45^ although some studies show an association with the AXIN2 gene in several cancers, including the ovarian one.^30^^,^^46^ The epidemiology of the epithelial ovarian cancer requires attention, because it is considered the fifth most common cause of cancer in women and the fourth leading cause of cancer death,^47^^,^^48^ with a prognosis of approximately 18 months for women with an advanced stage, and 40-50% of overall survival for all ovarian cancer at ten years.^49^ It is important the attempt of early establish the co-occurrence between the epithelial ovarian cancer and the dental agenesis as a risk factor, mainly because of the aggressiveness of this type of cancer, that is considered malignantly fatal and silent, therefore to the difficult of diagnosis,^21^^,^^50^ as the major symptoms are not specific^51^ and as the lack of effective screening markers.^46^ Some hypothesis that would be useful in the identification of ovarian cancer are to check the family history of this cancer^52^ and to identify tooth agenesis as a risk marker.^44^

### Limitations

Some aspects of this systematic review need further attention. Firstly, there were wide variations in the evaluation methods used to diagnose tooth agenesis in the studies. For future research, adequate and standardized methods of diagnoses and data collection are necessary. Secondly, small samples may not allow for statistically significant results. An issue related to the sample size was responsible for a fair quality in the study conducted by Paranjyothi, et al.^24^ (2018).

A possible genetic relationship between cancer and dental agenesis was suggested in the discussion, as genes involved in both conditions were reported. Nevertheless, the GRADE tool certainty of the evidence was classified as very low because of the observational study design and methodological flaws of the included articles. Thus, this subject needs to be studied more deeply, and a possible association should not be ruled out. More studies are needed, preferably prospective, to clarify the predictive value of tooth agenesis as an early indicator of cancer risk.

## Conclusions

Considering the limited scientific evidence, it is possible there is an association between dental agenesis and cancer. Tooth agenesis could then be an early indicator for cancer. Therefore, it is important for dentists to carefully observe cases of multiple agenesis in their offices and to indicate a more rigorous medical follow-up. Nevertheless, studies with a better level of evidence are needed to confirm this association.
